# Data-driven assessment of well stimulation in unconventional gas reservoirs

**DOI:** 10.1038/s41598-024-82454-z

**Published:** 2024-12-28

**Authors:** Jian Yang, Song Li, Ji Zeng, Zhaozhong Yang, Xiaogang Li, Tingting He, Liangping Yi, Bing Kong

**Affiliations:** 1 Egineering Research Institute of Petrochina Southwest Oil and Gas Field Company, Chengdu, 610017 China; 2 National Energy High-sour Gas Reservoir Exploitation and R & D Center, Guanghan, 618300 China; 3 School of Oil & Natural Gas Engineering, SouthWest Petroleum University, Chengdu, 610500 China; 4 State Key Laboratory of Oil and Gas Reservoir Geology and Exploitation, Chengdu, Sichuan 610500 China; 5 School of Mechanical and Electrical Engineering, SouthWest Petroleum University, Chengdu, 610500 China

**Keywords:** Unconventional gas reservoirs, Stimulation, Data-driven, Geological data, Hydraulic fracturing, Energy science and technology, Engineering

## Abstract

Unconventional gas reservoirs, characterized by their complex geologies and challenging extraction conditions, demand innovative approaches to enhance gas production and ensure economic viability. Well stimulation techniques, such as hydraulic fracturing and acidizing, have become indispensable tools in unlocking the potential of these tight formations. However, the effectiveness of these techniques can vary widely depending on the specific characteristics of the reservoir. In this context, a data-driven approach to assess well stimulation practices offers a promising avenue to optimize recovery processes and reduce uncertainties. This paper presents a comprehensive study that leverages the power of big data analytics and machine learning to analyze and improve well stimulation strategies in unconventional gas reservoirs. By systematically gathering and processing vast arrays of geological, operational, and production data, this study aims to identify patterns and correlations that can predict stimulation outcomes more accurately. The ultimate goal is to develop a robust framework that allows for tailored stimulation designs based on the unique properties of each reservoir, thereby maximizing efficiency and minimizing environmental impacts. This study introduces a new procedure for assessing well stimulation performance, which involves analyzing the EUR through Duong’s model, calculating the key performance indicator of the treatment, and establishing a data-driven model to predict the treatment KPI.

## Introduction

Unconventional gas reservoirs, characterized by their complex geologies and low permeabilities, require more advanced extraction techniques than conventional reservoirs to ensure economic viability and efficient gas production. Traditional extraction methods often fall short in these settings, making well stimulation techniques like hydraulic fracturing and acidizing crucial. These methods, however, exhibit varying degrees of effectiveness, heavily influenced by the geological characteristics of the reservoir^1^.

The literature on hydraulic fracturing reveals a significant focus on understanding the geomechanical properties of shale formations and their responses to stimulation techniques. According to Zhu et al.^2^, the microseismic events during fracking provide insights into fracture spread and efficacy, suggesting optimization strategies for fracture propagation. Guo et al.^3^ emphasize the optimization of fracturing fluid viscosity to improve conductivity and fracture propagation. Bandara et al.^4^ explore the influence of proppant characteristics on fracture permeability, demonstrating significant variations in long-term production rates based on proppant selection. Zhao^5^ discuss the role of pumping rates and pressures, noting that increased rates can expand fracture networks but also raise the risk of induced seismicity. Additionally, Zhang et al.^6^ highlight the importance of geological factors such as rock stiffness and in-situ stresses in dictating fracture behavior and effectiveness. Gong et al.^7^ investigate the impact of well orientation and depth on fracturing efficiency, illustrating that optimal alignment with stress fields can significantly affect production outcomes.

Conversely, acidizing is a critical well stimulation technique used to enhance permeability and production in both conventional and unconventional reservoirs, particularly in carbonate and sandstone formations. Research in this domain focuses on the chemical interactions between acid solutions and rock matrices, optimizing acid composition to maximize the dissolution of minerals and the creation of conductive flow paths. For instance, Martyushev et al.^8^ explore the effectiveness of acid treatments in carbonate reservoirs, identifying optimal acid concentrations that balance rock dissolution with structural integrity preservation. Ali et al.^9^ investigates the role of acid type and concentration in sandstone formations, demonstrating how specific acid blends can reduce formation damage caused by fines migration. Jiang et al.^10^ discuss the integration of thermochemical reactions in acidizing processes to improve the depth and uniformity of penetration in high-temperature wells. Additionally, these studies collectively advance the understanding of acidizing as a nuanced stimulation technique that requires careful consideration of geological, chemical, and environmental factors to optimize performance and sustainability in hydrocarbon extraction.

Recent advancements in big data analytics and machine learning have opened new avenues for optimizing well stimulation beyond traditional experimental and modeling approaches. Hsu et al.^11^ demonstrated the utility of machine learning in predicting fracture behavior from historical data sets, while Xiong et al.^12^ applied advanced analytics to optimize the composition and injection rates of fracturing fluids. This paper builds on these methodologies, proposing a comprehensive approach that synthesizes geological, operational, and production data to refine well stimulation strategies in unconventional gas reservoirs.

The cornerstone of this study is the development of a robust framework for tailored well stimulation, which is critical for maximizing operational efficiency and minimizing environmental impacts. We introduce a novel procedure for assessing well stimulation performance, utilizing the Duong’s model^13^. This model, augmented by machine learning techniques, enables the calculation of key performance indicators that predict treatment outcomes with greater accuracy. The literature underscores the importance of data-driven approaches in unconventional reservoir exploitation. Xiao et al.^14^ assert that the application of predictive analytics can significantly enhance the accuracy of stimulation outcome predictions. Moreover, Janković et al.^15^ discuss the environmental benefits of these approaches, demonstrating how optimized resource extraction processes can lead to reduced ecological footprints.

In conclusion, this paper not only seeks to advance the technical understanding and application of well stimulation techniques in unconventional gas reservoirs but also aims to contribute to the broader discourse on sustainable energy production practices. By integrating empirical research with cutting-edge data analytics and machine learning, this study provides actionable insights into the optimization of resource extraction, ensuring both economic feasibility and environmental sustainability. This approach ultimately enables the design of more efficient and environmentally friendly stimulation strategies that are customized to the unique properties of each reservoir.

## Methodology

This study’s methodology assesses the stimulation performance using the fracturing wells in Xujiahe and acidizing wells in Carboniferous as an example. It includes the following steps:

1. Data Preparation and Preprocessing.
  Feature Collection: Over 20 geology, drilling, and completion features were collected.
  Preprocessing: Cleaned data and conducted feature engineering and addressed multicollinearity by removing or transforming correlated features to ensure model integrity.
2. Decline Curve Modeling and EUR Prediction.
  Duong’s model is employed for decline curve modeling, which is well-suited for unconventional gas reservoirs. Duong’s model accounts for the prolonged transient flow periods typical in tight formations and the transition to boundary-dominated flow, providing a more accurate representation of production behavior in these reservoirs.
3. KPI Calculation from EUR.
  For acidized wells, since the wells have been in production prior to treatment, it is possible to calculate the EURs both before and after the treatment. The percentage improvement in EUR post-treatment is then used as a key performance indicator (KPI).
  For wells treated with hydraulic fracturing, the treatment is applied before the wells are connected to production infrastructure. Consequently, the EUR itself is utilized as the KPI for these wells.
4. Stimulation KPI Prediction Using Machine Learning.
  Feature Selection: Employed an empirical Bayesian Network (BN) for identifying features with causal relation and significant predictive power for stimulation performance.
  Stacked Modeling Framework: Utilized a two-tiered approach involving training of base models and a subsequent meta model with physical constricted feature values.
5. Feature Sensitivity Analysis.
  SHAP Value Analysis: Conducted to quantify the impact of features on stimulation performance, identifying key influencers.

## Data preparation and statistical analysis

In the field of unconventional reservoir exploitation, the production success critically depends on detailed analysis of geology, drilling, and completion data. This research concentrates on the Xujiahe and Carboniferous wells, where over 20 distinct features have been collected and preprocessed for machine learning modeling. The preprocessing involves transforming categorical features into sub-features using one-hot encoding, a technique that converts categorical data into a binary matrix. This is followed by addressing multicollinearity among the features, which is managed by either removing features with high levels of linear correlation or converting them into new, interaction-based features.

**Fig. 1:**
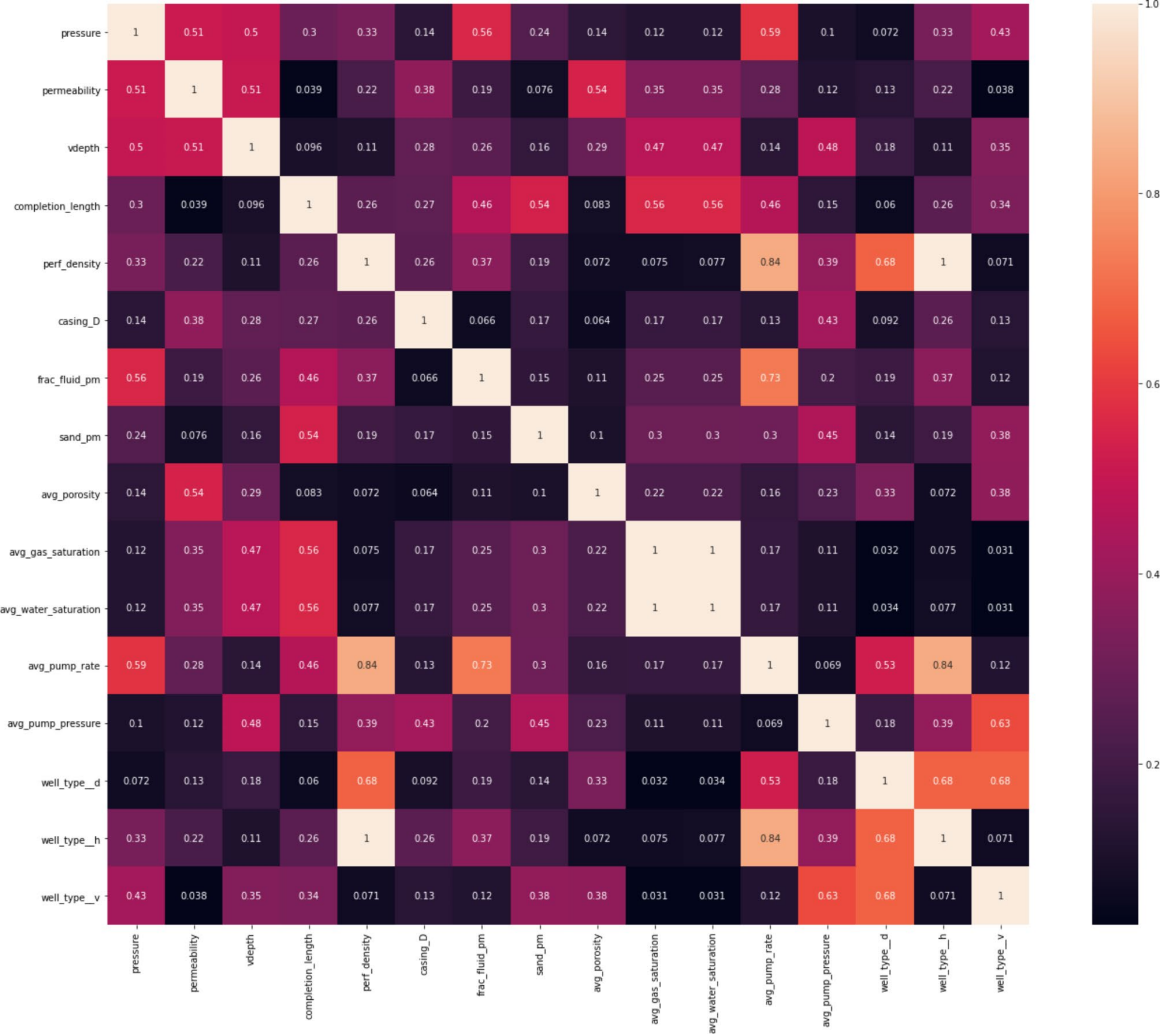
Correlation map of the model input features.

The correlation map produced (Fig. 1) post-preprocessing shows that the features exhibit low mutual correlations, indicating minimal redundancy. The most significant correlation, observed between porosity and carbonate volume percentage, remains below 0.6, suggesting a moderate relationship that does not compromise the integrity of the dataset. New interaction features introduced include normalized completion length by the number of stages, fracturing fluid volume, sand tonnage, and the number of perforations, adding valuable dimensions to the dataset by capturing complex interdependencies between drilling and completion variables.

The features are divided into three main categories: geology, drilling, and completion. This classification aids in organizing the analysis and underscores the comprehensive nature of the data influencing hydraulic fracturing performance. Table 1 presents a statistical summary of these features, including mean, standard deviation, and data range. This summary highlights the variability within the dataset and assists in recognizing patterns that may impact production.

**Table 1:** Descriptions of the model input features.

Measure	Count	Mean	Std	Min	25%	50%	75%	Max
Pressure	131	35.17	4.66	29.5	31.5	34.13	37.58	50.2
Permeability	104	0.09	0.06	0.01	0.05	0.1	0.1	0.42
Vdepth	147	2194.59	326.82	491.33	2215.15	2266.58	2320.86	2439
Completion_length	147	27.3	42.26	3	5	8	15.5	160
Perf_density	133	15.03	2.64	3	16	16	16	16
Casing_D	145	138.52	22.8	73	127	139.7	139.7	177.8
Frac_fluid_pm	147	43.02	47.98	0.83	13.06	27.73	55.43	326.25
Sand_pm	147	309.08	71.73	32.17	294.66	307.8	350	404.3
Avg_porosity	132	7.97	1.84	5	7	7.64	9	15
Avg_gas_saturation	133	62.23	11.06	15	56.8	62.5	70.65	87.5
Avg_water_saturation	133	37.11	11.02	12.5	28.95	36	41.5	85
Avg_pump_rate	147	5.63	3	3.25	3.75	4	6.5	12
Avg_pump_pressure	143	70.84	10.59	45	61	70	81	85
Cum_prod	147	1.74	1.66	0	0.28	1.52	2.72	6.64
Well_type_d	147	0.48	0.5	0	0	0	1	1
Well_type_h	147	0.35	0.48	0	0	0	1	1
Well_type_v	147	0.17	0.38	0	0	0	0	1

By integrating geology, drilling, and completion data in a machine learning framework, this study aims to identify key predictors of production performance in unconventional reservoirs. This approach demonstrates the necessity of a cross-disciplinary strategy to optimize production outcomes in complex geological settings. 

## Decline curve modeling and EUR prediction

### Duong’s rate decline model

Duong’s rate decline model^13^ is designed for unconventional reservoirs, where extended transient flow and delayed boundary-dominated flow are prevalent. In such reservoirs, the low permeability of the rock formation, combined with hydraulic fracturing, often leads to prolonged periods of transient flow before the reservoir reaches boundary-dominated conditions. Traditional models, such as Arps, are generally inadequate in these cases, as they assume an earlier onset of boundary-dominated flow, which can result in underestimating both production rates and the ultimate recovery.

Duong’s model addresses this limitation by incorporating a time-dependent exponent that adapts to the extended transient phase, making it more flexible and suitable for the unique production characteristics of tight formations. By capturing the gradual transition from transient to boundary-dominated flow, Duong’s model provides a more accurate framework for predicting the EUR in unconventional reservoirs. This is particularly important in shale gas and tight gas plays, where long-term production forecasts are critical for assessing economic viability and optimizing field development strategies.

In practice, Duong’s model is widely used for decline curve analysis in these types of reservoirs because it aligns more closely with actual production data compared to traditional models. Its ability to provide more reliable EUR estimates enhances decision-making regarding well placement, stimulation design, and reservoir management.

### Production decline curve analysis

**Fig. 2:**
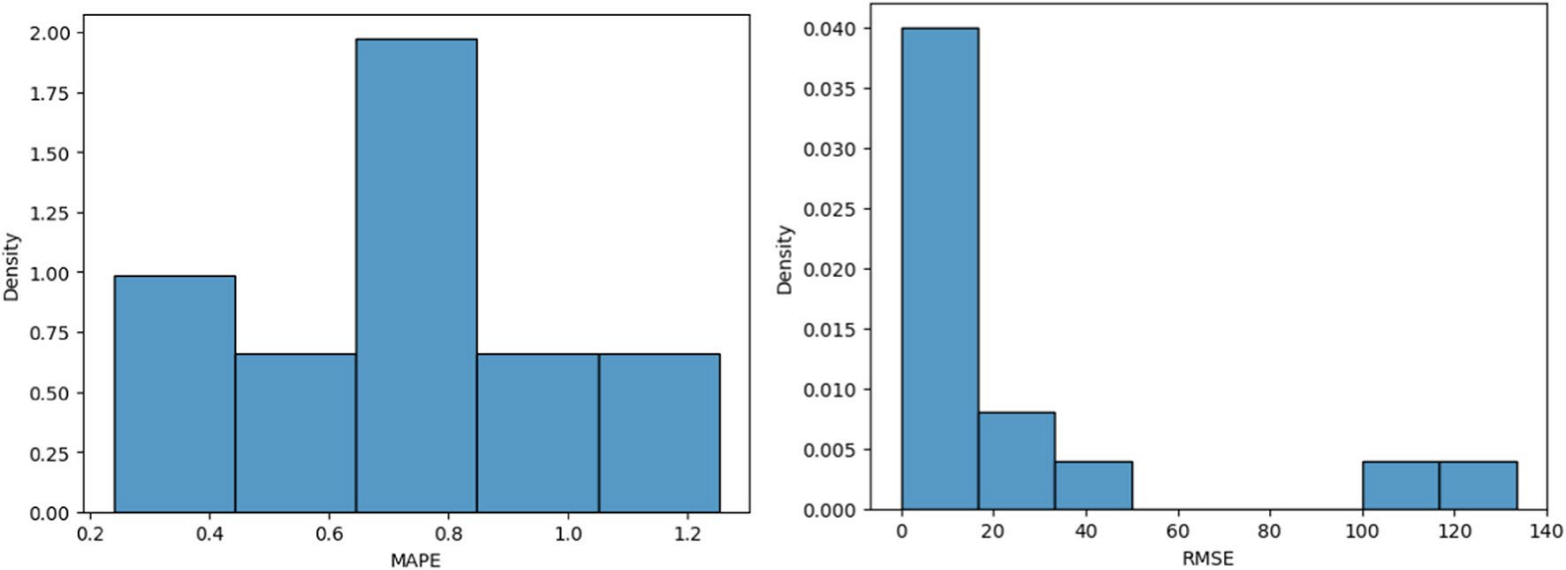
Error analysis of the simulations.

In the Xujiahe and Carboniferous region, there are totally 259 wells with valid production history when the data was collected. The production data including crude, condensate, and gas rate was gathered for each well. Because this play is producing both gas and oil, the production rates are converted to the oil of equivalent per day as the unified unit (1111 m^3^ gas to 1 ton of oil). The Duong’s model is applied to the production profile of each well to obtain the production decline parameters. The mean absolute percentage error (MAPE), root mean square error (RMSE) of the simulations are shown in Fig. 2 which demonstrating a strong fit of the model to the data.

**Fig. 3:**
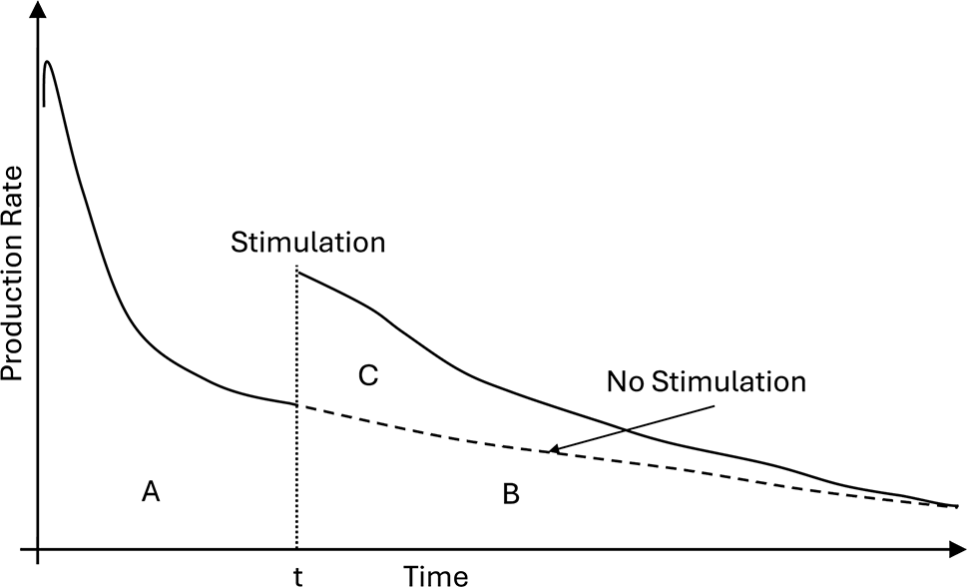
EUR distribution of the hydraulic fractured wells.

After the production decline curve analysis and obtaining the production decline parameter values of each well, the EURs of the wells in study are calculated. Figure 3 shows the aggregated EUR results, and it is shown that Xujiahe EUR follows a log-normal distribution, and the median is about 73000Kt.

## Stimulation performance assessment based on EUR

**Fig. 4:**
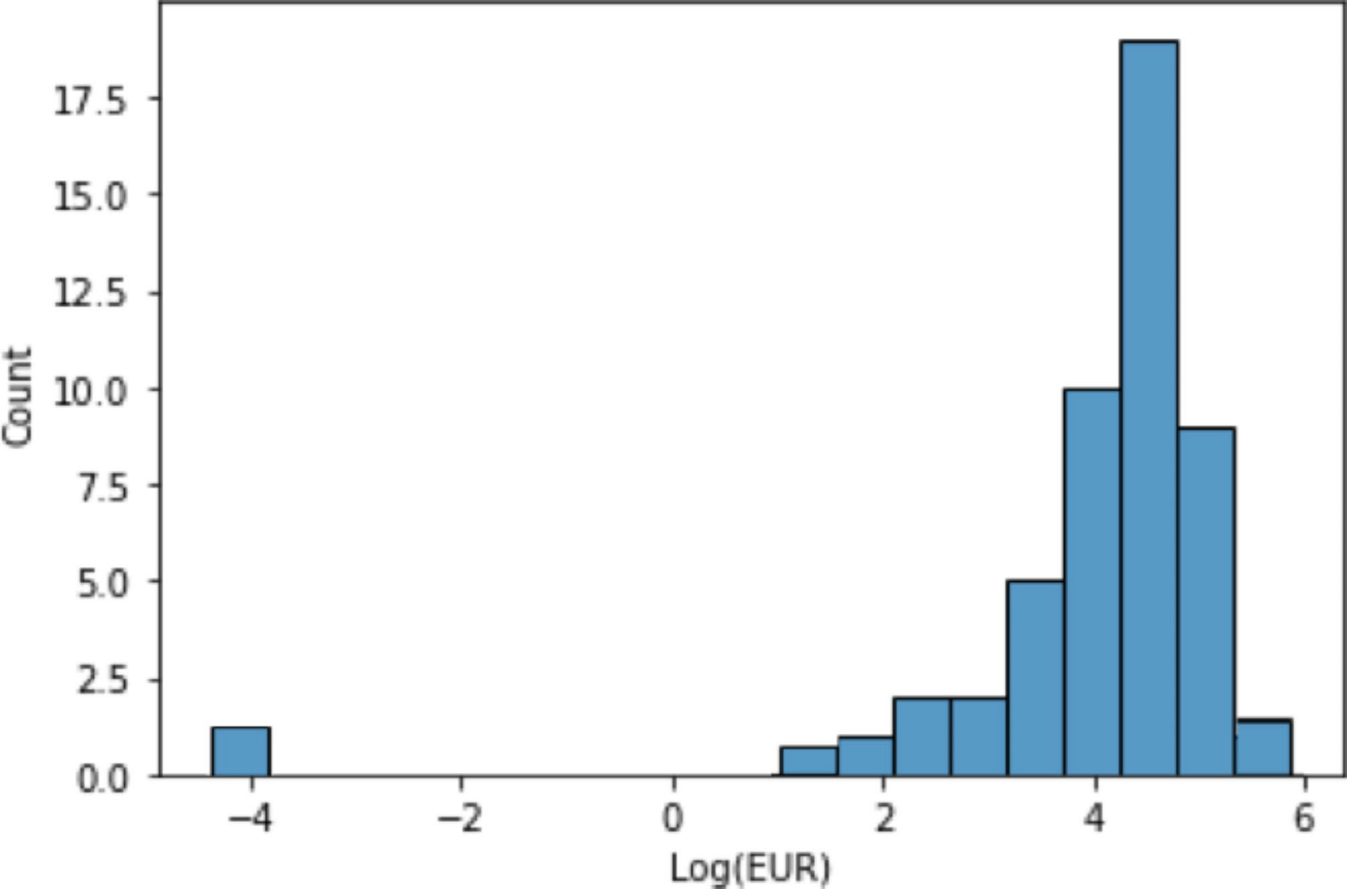
Stimulation Performance Evaluation Based on the EUR.

EUR measures the total volume of recoverable hydrocarbons, including natural gas, condensate oil, and crude oil, under specified conditions. In this study, EUR is utilized to assess stimulation performance in oil wells. For hydraulically fractured wells, stimulation treatment is performed prior to production, making EUR a critical performance indicator. Conversely, for wells treated with acid, stimulation usually occurs after initial production, so the percentage improvement in EUR serves as the primary performance metric. As depicted in Fig. 4, area A represents the cumulative production before stimulation. The sum of areas A and B constitutes the original EUR, calculated from production history before the stimulation at time t, while area C indicates the additional EUR gained from stimulation. The EUR improvement percentage is calculated as the ratio of area C to the sum of areas A and B. Area B is determined by subtracting area A from the original EUR, and area C by subtracting area B from the improved EUR post-stimulation.

**Fig. 5:**
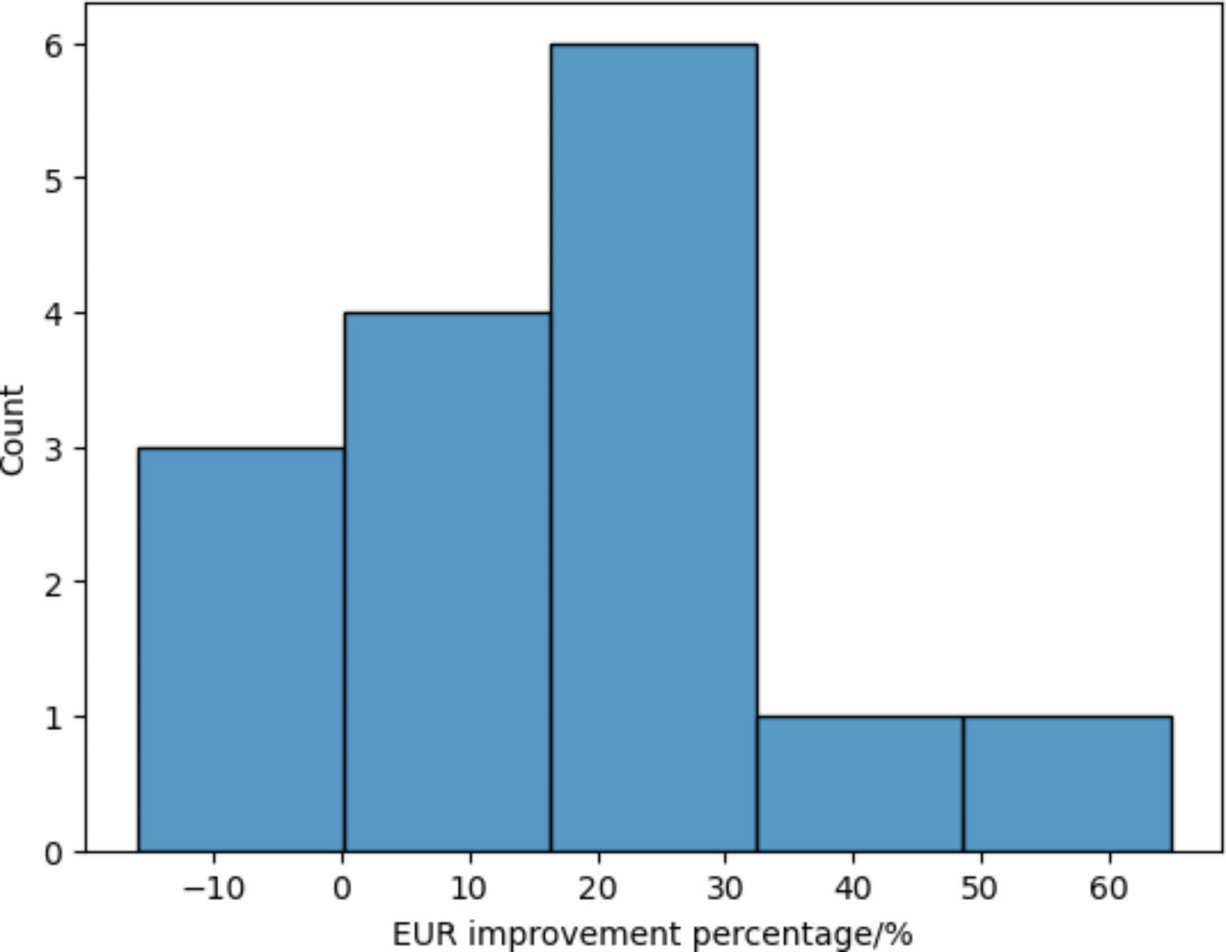
EUR improvement percentage of the acidized wells.

Using the methodology described in this study, the Estimated Ultimate Recoveries (EURs) of hydraulically fractured wells are calculated and displayed in Fig. 3. The EURs exhibit a log-normal distribution, centered around a value of 4.6. A small subset of these wells show very low EURs, indicative of unsuccessful stimulation. For the acidized wells, their EUR improvement percentages are presented in Fig. 5. The data reveals that most wells experience a 25% improvement in EUR post-stimulation, with some achieving increases up to 60%. However, some wells show a decrease in EUR following stimulation, which may be attributed to ineffective design or engineering failures.

## Data-driven stimulation performance prediction

### Stimulation performance modeling using machine learning

To effectively analyze and predict the stimulation performance, a data-driven approach that leverages information from similar wells is more practical than relying solely on Estimated Ultimate Recovery (EUR) methods. This strategy starts with feature engineering to refine the dataset, ensuring that only the most relevant variables are included for analysis.

**Fig. 6:**
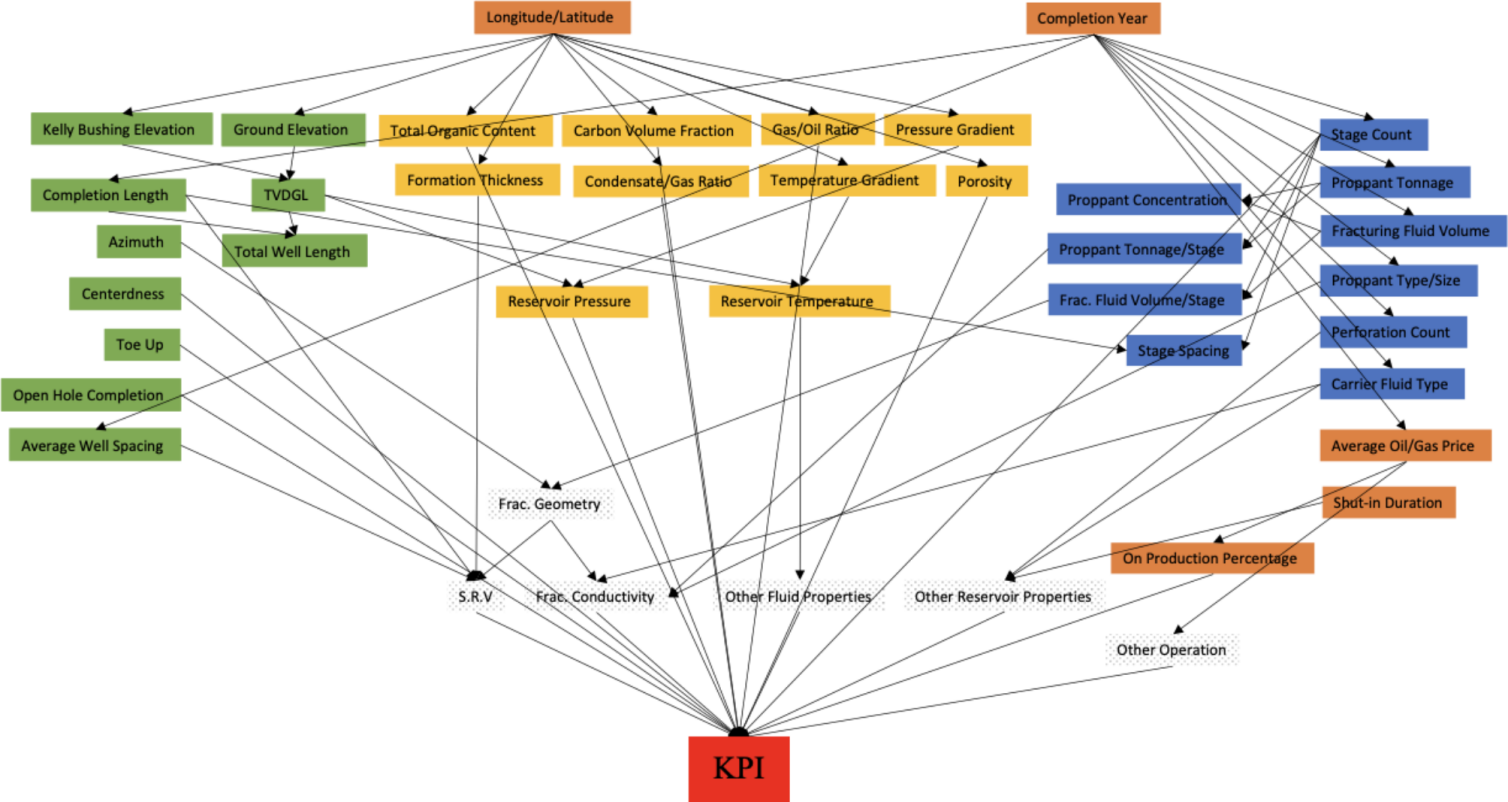
Bayesian Network of KPI of stimulation performance.

An empirical Bayesian Network (BN) is then used for feature selection, focusing on stimulation performance. This network, which encompasses the entire dataset as depicted in Fig. 6, pinpoints the key features that significantly influence the predictive power concerning stimulation performance. Employing a Bayesian Network in this context facilitates a more efficient machine learning training process by isolating the most pertinent features for predicting stimulation performance.

Once the relevant features for predicting the KPI of stimulation performance are identified through the feature selection process, the focus shifts to training machine learning models. Previous studies have shown that for a complex process with high dimension data and relatively small data size, traditional machine learning models such as neural network and tree-based models suffers from overfitting problem^16,17^. To overcome this limitation, a stacked model framework was adopted in this study. This approach involves a two-step process: training base models followed by training a meta model.

Stacked modeling, or stacking, is a sophisticated ensemble technique where multiple predictive models, such as decision trees, support vector machines, or neural networks, are first trained on the same dataset to generate predictions. These predictions are then used as inputs for a second-level model, known as the meta model. This meta model, often simpler, like a linear regression, integrates these varied predictions. Its key role is to synthesize them into a final output, effectively smoothing out individual model biases and errors to enhance overall accuracy^18^. The procedure starts with the training of various base models using the dataset, where each model undergoes hyperparameter optimization via a Bayesian optimization algorithm. This step, aimed at maximizing the model’s performance, is measured by a 10-fold cross-validation score, ensuring that each model is finely tuned to capture the underlying patterns and relationships in the data.

**Fig. 7:**
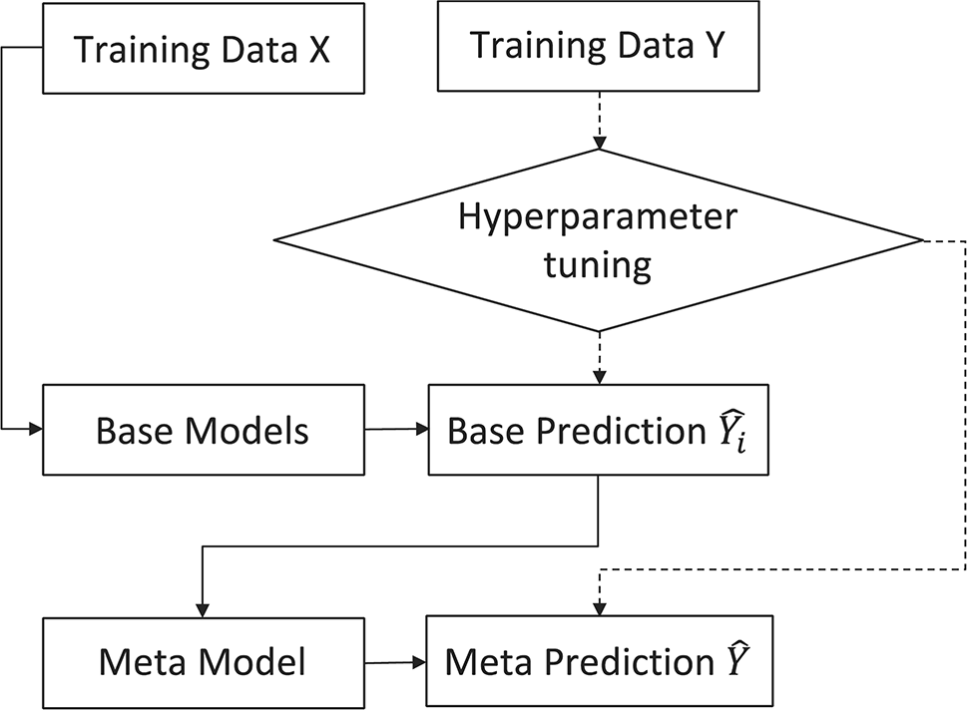
Modeling process.

After optimizing and training the base models, their predictions, along with the target variable from the training dataset, are used to train the meta model. This meta model, serving as the second layer in the stacked model architecture, is designed to learn how to optimally combine the predictions from the base models into a cohesive final prediction. This method not only leverages the strengths of each base model but also mitigates their individual weaknesses, enhancing the overall predictive accuracy. This modeling technique, illustrated in Fig. 7, utilizes the complementary capabilities of multiple machine learning models to achieve a high degree of accuracy in predicting stimulation performance.

Table 2 provides a comparative analysis of performance metrics for individual base models against the aggregated performance of the stacked model in predicting well stimulation performance. Notably, some base models, such as gradient boosting and the extra trees model, show strong training scores, which underscores their effectiveness at learning from the training dataset. However, these models also exhibit a significant performance drop on the testing dataset, suggesting a tendency to overfit. This is most likely caused by the limited data size of this study, which also justifies the adoption of staked model.

**Table 2:** Base model and meta model performance comparison.

model	Train *R*^2^	Test *R*^2^
Random forest	0.86	0.47
Lasso	0.41	0.33
Gradient boosting	0.96	0.56
XGBoost	0.95	0.59
HistGB	0.91	0.55
AdaBoost	0.77	0.46
Extra tree	0.83	0.45
Neural network	0.51	0.38
Stacking regressor	0.96	0.60

In contrast, the stacked model surpasses the individual base models, achieving the highest scores on both training and testing datasets. This superior performance underscores the stacked model’s ability to enhance overall predictive accuracy beyond the capabilities of any single model. By effectively combining predictions from various base models and mitigating their overfitting issues, the stacked model achieves a higher level of prediction accuracy and generalizability.

### Sensitivity analysis of stimulation performance

**Fig. 8:**
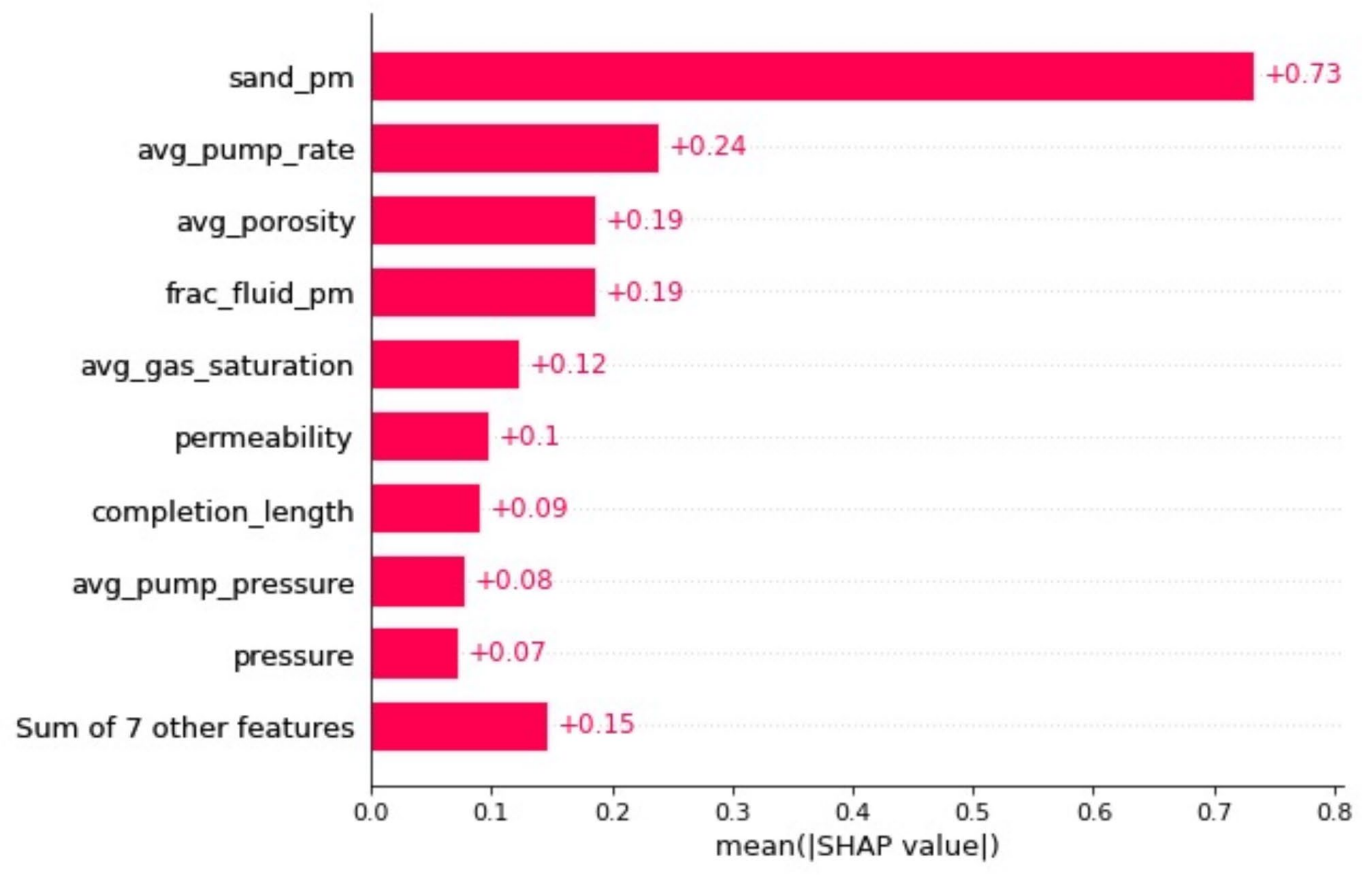
Fractured well EUR model feature importance measured by the mean absolute Shapley value.

**Fig. 9:**
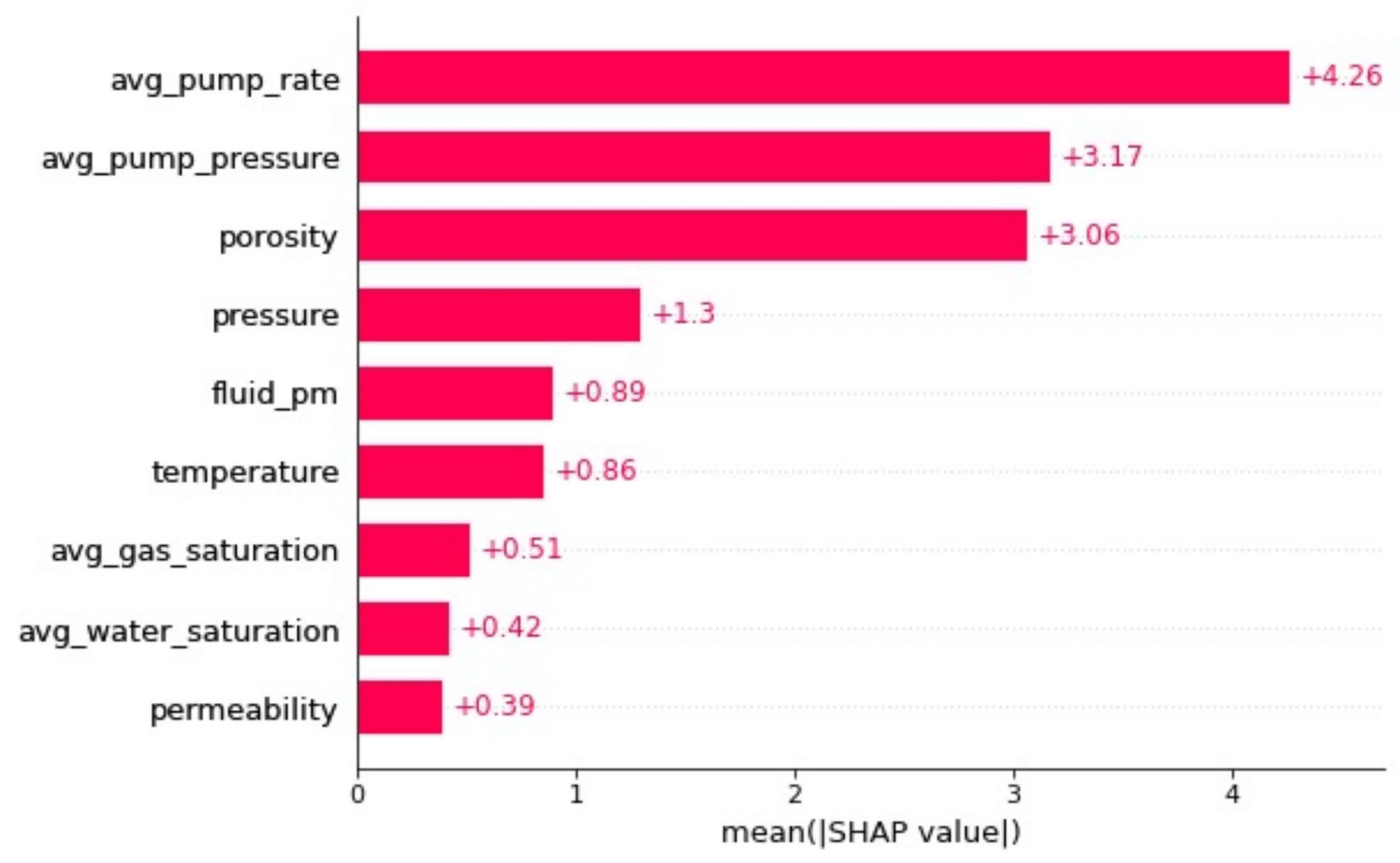
Acidized well EUR improvement percentage model feature importance measured by the mean absolute Shapley value.

After completing the training phase for the models, the influence of different features on the predicted outcomes is evaluated. SHAP (SHapley Additive exPlanations) and LIME (Local Interpretable Model-agnostic Explanations) analyses both offer valuable insights of the feature sensitivity. SHAP provides a global interpretation approach, attributing feature importance based on the cooperative game theory and ensuring consistency with the model output. It offers precise contributions of each feature to individual predictions, facilitating a deeper understanding of model behavior across the entire data set. LIME, on the other hand, focuses on local interpretability, approximating the predictions of the model locally around the prediction of interest. This method is particularly useful for explaining individual predictions but may vary depending on the locality and perturbations used. Since the aggregated global feature sensitivity is more meaningful in this study, SHAP is employed for the sensitivity analysis. This analysis is essential as it quantifies each feature’s contribution to the target variable. The ranking of feature importance, as determined by their mean absolute Shapley values, is illustrated in Figs. 8 and 9. The mean absolute Shapley value of a feature indicates its average impact on the model’s prediction, providing insights into which features are most influential.

The analysis shows that certain features, such as proppant per meter, average pump rate, average porosity, and fracturing fluid per meter, are particularly impactful on the performance of hydraulic fracturing treatments, significantly outweighing other features. This distribution of feature importance aligns with the fundamental factors that dictate the EUR of a well, primarily the quantity and quality of the resources within the stimulated reservoir volume.

Figure 9 focuses on the acidized wells, highlighting that the average pump rate, average pump pressure, porosity, and pressure are the most crucial features for the performance of acidizing. These findings underscore the significance of operational parameters and reservoir characteristics in optimizing well stimulation techniques.

## Summary

This paper explores the application of data-driven approaches in assessing well stimulation techniques for unconventional gas reservoirs. By integrating big data analytics and machine learning, the study aims to enhance the prediction and performance of well stimulation, crucial for increasing gas production efficiency and ensuring economic feasibility.

A novel methodology was developed to assess stimulation performance, utilizing the Duong’s model and a data-driven methodology to predict key performance indicators (KPIs) for different stimulation methods. For hydraulic fracturing, the treatment KPI is based on the EUR before connection to production infrastructure, whereas for acidized wells, the EUR improvement percentage post-treatment serves as the KPI.

The methodology encompasses data preparation and preprocessing, with a comprehensive collection of geological, operational, and production data. Feature selection was carried out using an empirical Bayesian Network, pinpointing critical predictors of well stimulation performance. The paper also introduces a stacked modeling framework for machine learning, which combines base model training with a meta model to refine predictions and address potential overfitting.

The study further evaluates feature sensitivity through Shapley value analysis, identifying key features that influence the performance of stimulation techniques. The results demonstrate that certain operational parameters and reservoir characteristics significantly affect the effectiveness of well stimulation, guiding the design of more efficient and tailored stimulation strategies.

In conclusion, the research highlights the importance of a cross-disciplinary, data-driven approach in tackling the challenges of unconventional reservoirs, proposing a robust framework that not only predicts but also improves the outcomes of well stimulation practices. This approach ultimately contributes to more efficient resource recovery and reduced environmental impacts in the oil and gas industry.

## Data Availability

The data that support the findings of this study are available from the corresponding author upon reasonable request.
